# The Effect of Authentic Leadership on Nurses’ Trust in Managers and Job Performance: A Cross-Sectional Study

**DOI:** 10.3390/nursrep12040095

**Published:** 2022-12-09

**Authors:** Bayan Alilyyani

**Affiliations:** Nursing Department, College of Applied Medical Sciences, Taif University, P.O. Box 11099, Taif 21944, Saudi Arabia; b.alilyyani@tu.edu.sa; Tel.: +966-541012668

**Keywords:** leadership, authentic leadership, trust in managers, job performance, nursing, quantitative

## Abstract

Background: Nurse leaders have the responsibility to build healthy work environments for staff nurses and enhance nurses’ outcomes. Authentic leadership is one of the leadership theories that have been shown to have positive impacts on nurses’ outcomes. The goal of this study was to test the effect of authentic leadership on trust in managers and job performance among nurses in Saudi Arabia. Methods: A non-experimental, cross-sectional design was applied. A total of 116 nurses who met the inclusion criteria completed the survey. To test the study variables, three different scales were used. The data in this study were analyzed using SPSS version 28.0.1.1. Results: The findings of this study showed that there were significant and positive effects of authentic leadership and its four components on trust in managers. However, no relationships were found between authentic leadership and its four elements, and job performance. Conclusions: Authentic leaders have the ability to improve work environments by building a trustful relationship with nurses. This study focuses on the role of authentic leadership in nursing practice and its essential effects to enhance the work environments. It also provides future researchers in Saudi Arabia with comprehensive knowledge about conducting studies of authentic leadership in nursing and examine its effects on outcomes related to nurses.

## 1. Introduction

Nursing faces different current challenges, especially during pandemics. These challenges can affect nurses’ job performance, which in turn can affect the quality of nursing care, patient or nurse satisfaction, and the quality of health organizations’ services [1,2]. Therefore, these complex challenges in health care and specifically in nursing require the presence of efficient leaders/managers [3,4]. Additionally, the complexity of nurses’ duties itself requires complex leadership skills that can fit any situation in nursing practice [5,6]. Leaders have the responsibility to make an environment where nurses can adapt to any challenge. It was found that building a good relationship between leaders and followers helps to create a motivational and trustful environment where nurses can be confident in their actions and decisions [7].

Research has been focused on the trust concept and has paid more attention to it and its effect on organizations in different fields [8,9] and on the nursing profession more specifically [10]. Trust is one of the most essential aspects among individuals, so the presence of trust in any organization could help to enhance its outcomes. Building trust between employees and their supervisors also helps to provide healthy work environments [11]. In addition, nurse leaders have an essential impact on staff performance and on building a trustful relationship that ensures that effective and safe care is provided to all patients [12]. Trust in leaders can minimize organizations’ costs and maximize staff motivation, satisfaction, and performance [13]. Effective leaders have the ability to improve the quality of work and increase staff confidence in nurse managers and leaders [14].

Leadership is the process of influencing followers to achieve organizational goals using different leadership styles [15]. It is essential for nurse leaders to be aware of the different leadership styles in order to improve their leadership skills [16,17]. One of the leadership styles that has been recently used by nurse managers and leaders is authentic leadership. Authentic leadership positively influences self-awareness and self-regulation on the part of both leaders and followers [18]. Authentic leadership and its four elements, which are self-awareness, relational transparency, balanced processing, and internalized moral perspective, emphasize leaders’ insight, transparency, and congruence in their actions and beliefs [18]. Leaders who are authentic can make a significant difference in their organizations by helping staff to find meaning in their work, building optimism and trust among followers through transparent relationships, and providing a positive and healthy work environment [19]. They also focus on performing their tasks with ethical standards and make positive changes in others who work with them [20].

Authentic leaders are those who motivate their staff and build confidence and trust [21,22]. Authentic leadership was found to be an essential aspect that could improve organizational behaviors of staff [23], build ethical environments [24], enhance organizational commitment [25], maximize engaging staff in their work [26,27], improve the job performance of staff [28], and build trust between leaders and staff [27,29]. Authentic leaders can achieve high levels of authenticity because they are aware of their values and use them as guides in their work [20]. According to Avolio et al. [20], authentic leaders facilitate higher-quality relationships that motivate staff to be actively engaged in their work, enhance their satisfaction, and increase their productivity and performance.

Thus, nurses whose leaders are authentic show better job performance and are more likely to have a trustful relationship with their leaders or managers. Although trust and job performance are essential factors in nursing practice that can positively or negatively affect all nurses, patients, and the organization’s outcomes, a few studies have examined them and their relationship with leadership styles, more specifically the authentic leadership style. The aim of this study was to test the influence of authentic leadership on trust in managers and job performance among nurses in Saudi Arabia. The theoretical framework that was used to guide the study and the methods of the study are explained in the following sections.

### 1.1. Theoretical Framework

This study was guided by the authentic leadership theory [20]. The theory was developed to analyze the foundation of all previous leadership theories, such as transformational, charismatic, and emotional intelligence leadership [20,30,31]. Authentic leadership is defined as a pattern of a leader’s behavior that both builds upon and promotes “positive psychological capacities and a positive ethical climate, to foster greater self-awareness, an internalized moral perspective, balanced processing of information, and relational transparency on the part of leaders working with followers, fostering positive self-development” [18] (p. 94). The theory of authentic leadership explains the effect of authentic leadership and its four elements, which are balanced processing, relational transparency, internalized moral perspective, and self-awareness, on followers’ attitudes and behaviors through personal and social identification, hope, positive emotions, optimism, and trust [20].

Authentic leaders have the ability to enhance the work environment through four key components, which are balanced processing, relational transparency, internalized moral perspective, and self-awareness. Self-awareness is defined as the way in which an individual understands the world and makes meaning that reflects their views over time [32]. Relational transparency is related to involving others in making decisions and sharing information [32]. Balanced processing is when a person is able to objectively analyze all the relevant information before making any decision [33]. Internalized moral perspective refers to self-regulation, which can be guided by moral standards and values [18]. The authentic leadership theory of Avolio et al.’s [20] proposes that authentic leaders facilitate their followers via personal identification with leaders and social identification with the group and organization.

### 1.2. Hypothesized Model

The focus of authentic leadership is on the relationships between leaders and their followers [20]. Although there are different leadership theories that emphasize the behaviors and characteristics of leaders, only a few leadership theories focus on building relationships between leaders and their followers [21]. Authentic leaders are those who have high moral standards and values, which help followers to develop high and positive expectations about their leaders [20]. In addition, followers whose leaders are authentic have a high level of trust, because trust is one of the most essential moral standards of authentic leaders [20]. To enhance and build trust in leaders, authentic leadership should be applied in organizations [27]. Previous studies in nursing and other professions have suggested that authentic leadership plays an essential role in building a trustful relationship between leaders and followers [27,28,29]. More specifically, authentic leadership shows to have positive impacts on nurses’ trust in managers. Based on these findings, the following hypothesis was proposed:

**Hypothesis 1.**  *Authentic leadership and its four elements, which are transparency, balanced processing, moral/ethical perspective, and self-awareness, have a positive and significant effect on trust in managers*.

Authentic leadership was found to have an influence on followers’ performance and behaviors through improving trust in leaders and identification with leaders [18,20]. Authentic leaders can promote the positive attitudes and behaviors in their followers that could contribute to enhancing their job performance [34]. The four elements of authentic leadership, which are transparency, balanced processing, moral/ethical perspective, and self-awareness, can influence the followers’ performance [18,19,20,21]. Moreover, authentic leaders objectively analyze all the relevant information before making any decision and have followers involved in these decisions by asking them to share their point of view, which can be used to support their decisions [18]. As a result, staff whose leaders are authentic become more confident in their abilities, and they can perform well in their work [34]. Authentic leadership could show to have a positive impact on nurses’ job performance. Thus, the following hypothesis was proposed:

**Hypothesis 2.**  *Authentic leadership and its four elements, which are transparency, balanced processing, moral/ethical perspective, and self-awareness, positively and significantly influence job performance*.

**Figure 1:**
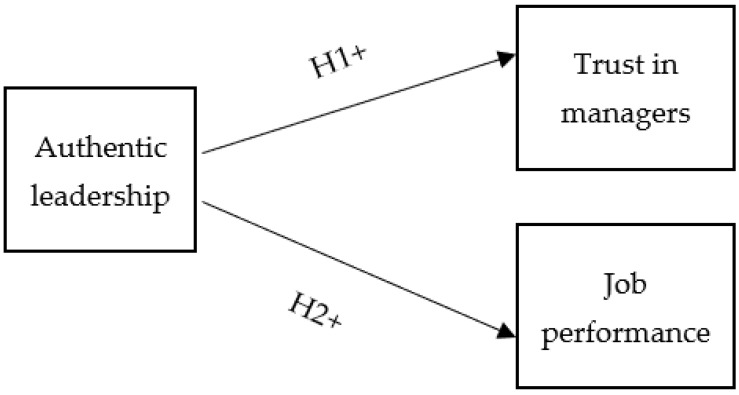
The hypothesized model.

Figure 1 illustrates the hypothesized model of the study. 

## 2. Materials and Methods

### 2.1. Design

A non-experimental, cross-sectional design was used to test the model. This study is compliant with Strengthening the reporting of observational studies in epidemiology (STROBE) [35].

### 2.2. Setting

This study was conducted in inpatient or outpatient departments in the selected hospital in Taif City, Saudi Arabia. The hospital is public and operated by the Saudi Ministry of Health.

### 2.3. Sample

Convenience sampling was used to select participants in this study. An online survey was sent to all nurses working in the selected hospital. A total of 116 out of 300 nurses completed the survey. Only nurses who were formally registered in Saudi Arabia, worked in the hospital departments, had six months or more of experience in their current departments, and agreed to voluntarily participating in the study were included in the study. Nurses who had less than six months of experience in their departments and were in any leadership or management position were excluded. Data were collected between May and July 2022.

### 2.4. Instruments

In this study, three different scales were used to measure the study variables. Authentic leadership was measured using the authentic leadership questionnaire, which consists, overall, of 16 items divided into four subscales (5 items for transparency, 4 items for internalized moral perspective, 3 items for balanced processing, 4 items for self-awareness. Items are rated on a 5-point Likert scale ranging from 0 (not at all) to 4 (frequently, if not always). The overall score of authentic leadership was the average of all items of the four subscales [36], so the highest score represented the highest authentic leadership rating. Examples of the items are “My leader says exactly what he or she means” and “My leader admits mistakes when they are made”. The reliability and validity of the scale were tested in previous studies [18].

To measure trust in managers, we used a scale consisting of 7 items rated on a 5-point Likert scale ranging from 1 (strongly disagree) to 5 (strongly agree) [37]. The average of all items represented the score of trust in managers, and a higher score meant higher trust in managers. The reliability and validity of the scale were measured by Norman et al. [37]. An example of the items is “I believe that my immediate supervisor/manager will keep his/her word.”

Job performance was measured using an instrument containing 9 items rated on a 5-point Likert scale ranging from 1 (strongly disagree) to 5 (strongly agree) [38]. The score of job performance was obtained by calculating the average of all items. A higher score represented higher job performance. The scale consisted of three reversed items. Examples of the items of the scale are “I am currently working at my best performance level” and “It is my right to use all my sick leave allowance (R).” Reliability and validity were tested and found to be acceptable in previous studies [38].

In addition to the three scales, a demographic questionnaire was used to collect information about participants, such as age, sex, nationality, the highest level of education, years of experience as registered nurses, and current department.

### 2.5. Data Analysis

Data were analyzed using SPSS version 28.0.1.1 (SPSS Inc., Chicago, IL, USA). A descriptive analysis was used to test the demographics of the participants. Means and standard deviations were used to analyze the average responses of the main study variables. Cronbach’s alpha was used to measure the reliability of the scales used in the study. Pearson correlation was used to measure the correlations among the main study variables. To test the study hypotheses, multiple linear regression was applied. Since there were only missing data in two responses in the age category, they were removed from the analysis to avoid bias.

### 2.6. Ethical Approval

Nurses were asked about their agreement to participate in the study before starting the online survey. In addition, participants were provided all the information about the study, and they voluntarily participated in the study. Responses were anonymous, so no identifying information was collected. Ethical approval for the study was obtained from the participating hospital located in Taif City, Saudi Arabia.

## 3. Results

### 3.1. Descriptive Results

The results of participants’ demographic data are illustrated in Table 1. All participants were female (100%) with a mean age of 37 years (SD = 8.39). The mean of nurses’ experience as registered nurses was around 14 years (SD = 7.99). Only 24% of nurses were Saudi, while the other were non-Saudi, of other nationalities. Most nurses had a bachelor’s degree in nursing as their highest education. The participating nurses were from different departments in the selected hospital, such as medical (28%) and pediatric intensive care unit (18%). There were only missing data in two responses regarding age, which were removed.

### 3.2. Means, Standard Deviations, and Reliability Analysis of the Study Variables

The results of the study variables’ means were considered as moderate to high. The overall mean of authentic leadership was 2.68 (*SD* = 0.86); we also calculated the means of its subscales, i.e., transparency (*M* = 2.69, *SD* = 0.86), balanced processing (*M* = 2.70, *SD* = 0.91), moral/ethical perspective (*M* = 2.67, *SD* = 0.93), and self-awareness (*M* = 2.65, *SD* = 0.99). The means of the outcome variables were calculated: trust in managers (*M* = 3.53, *SD* = 0.76) and job performance (*M* = 3.71, *SD* = 0.48). In addition, the results of Cronbach’s alpha, which represented the reliability of the scales of all three variables, were very good (overall authentic leadership, 0.87; trust in managers, 0.92; job performance, 0.94). Table 2 explains these results in detail.

### 3.3. Relationships among the Study Variables

**Figure 2:**
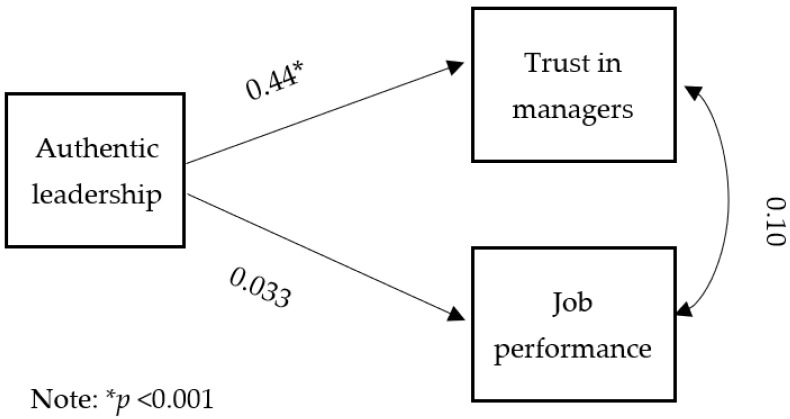
Results of hypothesized model.

The hypothesized model was tested using multiple linear regression Figure 2 illustrates the results of the hypothesized model. It was found that authentic leadership significantly and positively affected trust in managers (*R* = 0.44, *F* (1114) = 27.8, *p* < 0.001). In addition, there were and significant and positive relationships between all the four subscales of authentic leadership and trust in managers as follows: transparency, *R* = 0.38, *F* (1114) = 20.2, *p* < 0.001; balanced processing, *R* = 0.46, *F* (1114) = 30.9, *p* < 0.001; moral/ethical perspective, *R* = 0.39, *F* (1114) = 21.3, *p* < 0.001; self-awareness, *R* = 0.43, *F* (1114) = 26.6, *p* < 0.001. Thus, these results supported study Hypothesis 1.

It was found that there were no significant relationships between authentic leadership and job performance (*R* = 0.033, *F* (1114) = 0.12, *p* = 0.72). Additionally, no significant relationships were found between the four subscales of authentic leadership and performance: transparency, *R* = 0.003, *F* (1114) = 0.001, *p* = 0.97; balanced processing, *R* = 0.05, *F* (1114) = 0.35, *p* = 0.55; moral/ethical perspective, *R* = 0.04, *F* (1114) = 0.21, *p* = 0.64; self-awareness, *R* = 0.03, *F* (1114) = 0.13, *p* = 0.71. The second hypothesis, therefore, was not supported. Table 3 shows the results of the study variables’ relationships. 

The results did not support the relationship between trust in managers and job performance either (*r* = 0.10, *p* = 0.24). The range of correlations among the study variables was 0.003–0.95, as shown in Table 4.

## 4. Discussion

This study was conducted to examine the effect of authentic leadership on nurses’ trust in managers and job performance. The demographics showed that all the nurses that participated in this study were female (100%), which is in line with previous studies performed in Saudi Arabia that found that most of the participants were female nurses (88.5% [39] and 92% [40]). Most nurses were non-Saudi (76%) and held a bachelor’s degree in nursing (74%). Previous studies also showed that most nurses held bachelor’s degrees in nursing (75.4%) [41], and 69% of nurses were international nurses [42].

As hypothesized in Hypothesis 1, there were significant and positive relationships between authentic leadership and its four components, which are transparency, balanced processing, moral/ethical perspective, and self-awareness, and trust in managers. Thus, the first hypothesis was supported. Although there are a few studies that examined the relationships between authentic leadership and trust in managers in nursing [43,44,45], these results are aligned with the current study as they found that authentic leadership had a significant and positive relationship with trust in managers. The findings of this study and previous studies support that leaders who are authentic are more likely to build trust in their staff. In addition, leaders have the ability to develop a trustful environment in their organizations by showing honesty and truthfulness and encouraging nurses to build it as the norm and value of the organization [10]. They can also share their belief in trust with their staff, which in turn can improve the level of trust in staff as well as the organization [10].

There are studies conducted in other professions, such as business [27,46] and human resources [47]. The findings of these studies showed that authentic leadership positively and significantly influenced trust in managers ((*r* = 0.74, *p* < 0.01) [27], (*r* = 0.511, *p* < 0.01) [46], and (*r* = 0.725, *p* < 0.01) [47]).

On the other hand, the current results did not support the relationships between authentic leadership, composed of transparency, balanced processing, moral/ethical perspective, and self-awareness, and job performance. Therefore, the second hypothesis was rejected. A previous study was performed to examine the relationships between authentic leadership and structural empowerment, performance, and job satisfaction among 600 nurses in Canada [48]. Their findings did support the indirect relationship between authentic leadership and job performance through empowerment, but there was no direct correlation between authentic leadership and job performance [48]. These results are consistent with the current study, which found no relationship between authentic leadership and job performance. Another study was conducted to explore the effect of authentic leadership on trust in management and different work outcomes among clinical providers and nonclinical employees [49]. They found no relationship between the four elements of authentic leadership and job performance among clinical healthcare providers [49]. Thus, the results of the current study support these findings, so no relationships were found between the four components of authentic leadership and job performance. However, a study was performed to illustrate the relationship between authentic leadership and contextual performance among nurses and found that authentic leadership was significantly and positively related to contextual performance (*B* = 0.4379, *p* < 0.0000) [50], which is not consistent with the results of the current study.

In other professions, such as business, it was found that authentic leadership had a significant and positive influence on performance (0.11, *p* <0 .01) [51]; (0.19, *p* <0 .01) [34], which does not support the result of the current study.

### Implications for Nursing Practice and Research

The results of this study highlight the important role of authentic leadership in nursing practice, especially its effect on building trustful relationships with staff nurses. Leaders should be aware of their leadership styles and apply a suitable style in their nursing practice. It is also essential for nurse leaders to build trust in their workplace by applying the authentic leadership style, as it has been found to have an impact on nurses’ trust in their leaders. The results of this study support the fact that if nurses trust their leaders, they can feel engaged and satisfied in their work, which enhances patients’ outcomes [52]. Additionally, the results emphasize the need for future research to explore the influence of authentic leadership on trust in managers and job performance, as it was noticed that only few studies in nursing have examined these relationships. In Saudi Arabia, we need more studies focusing on authentic leadership and its effects on nurses’ outcomes [16].

There are some limitations to this study. This study used a cross-sectional design, which prevented the causality among the study variables. In addition, this study was conducted in one setting, which affects the generalizability of the study results, which are not applicable to all hospitals and nurses in Saudi Arabia. Another limitation was the sample size; it was a small size, which may have affected results from being extrapolated. Data were collected online using self-reported surveys, which could have affected the results’ bias.

## 5. Conclusions

The study emphasizes the essential impact of authentic leadership on nurses’ outcomes, which could have an influence on patients’ outcomes. Authentic leaders have the ability to enhance the work environment through building a trustful relationship with their staff nurses, which is considered to be a critical element in nursing practice. This study also provides comprehensive knowledge about applying authentic leadership in nursing practice and its effects on nurses’ outcomes, which could be explored by future researchers in Saudi Arabia. There is a gap in the nursing literature related to the influences of authentic leadership in nursing, more specifically in Saudi Arabia, which emphasizes future research to focus on exploring its effects which could help to improve nursing practice. 

## Figures and Tables

**Table 1:** Characteristics of participants’ demographics (*N* = 116).

Demographic Characteristic	*n*	%
Nationality	116	100%
Filipino	52	44.8%
Saudi	28	24.1%
Indian	28	24.1%
Indonesian	3	2.6%
Pakistani	3	2.6%
Malaysian	2	1.7%
Gender	116	100%
Female	116	100%
Male	0	0
Highest level of education	116	100%
Bachelor’s in nursing	88	74.1%
Diploma in nursing	28	24.1%
Master’s in nursing	2	1.7%
Current area	116	100%
Neonatal intensive care unit	51	44%
Medical	32	27.6%
Pediatric intensive care unit	21	18.1%
Isolation	3	2.6%
Education	3	2.6%
Cardiac	1	0.9%
Outpatient	1	0.9%
	M	SD
Age	*37.3*	*8.39*
Years of experience as registered nurses	*13.5*	*7.99*

**Table 2:** Means, standard deviations, and reliability analysis of study variables.

Study Variables	Response Range	Number of Items	*M*	*SD*	Cronbach’s Alpha
Authentic leadership	0–4	16	2.68	0.86	0.87
Subscales:					
-Transparency	0–4	5	2.69	0.86	0.88
-Balanced processing	0–4	3	2.70	0.91	0.88
-Moral/ethical perspective	0–4	4	2.67	0.93	0.88
-Self-awareness	0–4	4	3.53	0.76	0.87
Trust in managers	1–5	7	3.53	0.76	0.92
Job performance	1–5	9	3.71	0.48	0.94

**Table 3:** Relationships among study variables.

Study Variables	*R*	*F*	*df*	*p*
Authentic leadership 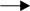 Trust in managers	0.44	27.8	1114	<0.001
Transparency 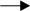 Trust in managers	0.38	20.2	1114	<0.001
Moral/ethical perspective 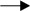 Trust in managers	0.39	21.3	1114	<0.001
Balanced processing 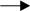 Trust in managers	0.46	30.9	1114	<0.001
Self-awareness 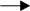 Trust in managers	0.43	26.6	1114	<0.001
Authentic leadership 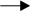 Job performance	0.033	0.12	1114	0.72
Transparency 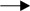 Job performance	0.003	0.001	1114	0.97
Moral/ethical perspective 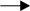 Job performance	0.04	0.21	1114	0.64
Balanced processing 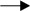 Job performance	0.05	0.35	1114	0.55
Self-awareness 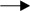 Job performance	0.03	0.13	1114	0.71

**Table 4:** Correlation matrix of study variables.

Variable	1	2	3	4	5	6
1. Authentic leadership	—	—	—	—	—	—
2. Transparency	0.940 *	—	—	—	—	—
3. Moral/ethical perspective	0.948 *	0.877 *	—	—	—	—
4. Balanced processing	0.915 *	0.783 *	0.815 *	—	—	—
5. Self-awareness	0.951 *	0.833 *	0.856 *	0.892 *	—	—
6. Trust in managers	0.443 *	0.388 *	0.397 *	0.462 *	0.435 *	—
7. Job performance	0.033	0.003	0.043	0.056	0.034	0.109

Note: * *p* < 0.001.

## Data Availability

The data presented in this study are available upon request from the corresponding author. The data are not publicly available due to privacy.

## References

[1] Pishgooie A.H., Atashzadeh-Shoorideh F., Falcó-Pegueroles A., Lotfi Z. (2019). Correlation between nursing managers’ leadership styles and nurses’ job stress and anticipated turnover. J. Nurs. Manag..

[2] Yau S.Y., Xiao X.Y., Lee L.Y., Tsang A.Y., Wong S.L., Wong K.F. (2012). Job stress among nurses in China. Appl. Nurs. Res..

[3] Prestia A.S. (2020). The Moral Obligation of Nurse Leaders: COVID-19. Nurs. Lead.

[4] Casida J., Parker J. (2011). Staff nurse perceptions of nurse manager leadership styles and outcomes. J. Nurs. Manag..

[5] Lamb A., Martin-Misener R., Bryant-Lukosius D., Latimer M. (2018). Describing the leadership capabilities of advanced practice nurses using a qualitative descriptive study. Nurs. Open.

[6] Laschinger H.K.S., Wong C.A., Cummings G.G., Grau A.L. (2014). Resonant leadership and workplace empowerment: The value of positive organizational cultures in reducing workplace incivility. Nurs. Econ..

[7] Specchia M.L., Cozzolino M.R., Carini E., Di Pilla A., Galletti C., Ricciardi W., Damiani G. (2021). Leadership styles and nurses’ job satisfaction. Results of a systematic review. Int. J. Environ. Res. Public Health.

[8] Kim T.Y., Wang J., Chen J. (2018). Mutual trust between leader and subordinate and employee outcomes. JBus Ethics.

[9] Soltani S. (2017). Investigating the relationship between different dimensions of organizational trust and organizational commitment of personnel. Educ. Manag. Innov..

[10] Hadi-Moghaddam M., Karimollahi M., Aghamohammadi M. (2021). Nurses’ trust in managers and its relationship with nurses’ performance behaviors: A descriptive-correlational study. BMC Nurs..

[11] Jazaei N., Soltani S. (2017). The effect of organizational justice on organizational commitment in the light of organizational trust. J. Dev. Manag..

[12] Basit G., Duygulu S. (2017). Nurses’ organizational trust and intention to continue working at hospitals in Turkey. Collegian.

[13] Shams S., Esfandiari M.A. (2015). Relationship between different dimensions of organizational trust and employee’s job satisfaction. J. Manag. Stud..

[14] Wilson A.A. (2005). Impact of management development on nurse retention. Nurs. Adm. Q..

[15] Leggat S.G., Balding C., Schiftan D. (2015). Developing clinical leaders: The impact of an action learning mentoring programme for advanced practice nurses. J. Clin. Nurs..

[16] Alilyyani B., Kerr M.S., Wong C., Wazqar D.Y. (2022). An integrative review of nursing leadership in Saudi Arabia. Nurs. Open.

[17] Madathil R., Heck N.C., Schuldberg D. (2014). Burnout in psychiatric nursing: Examining the interplay of autonomy, leadership style, and depressive symptoms. Arch. Psychiatr. Nurs..

[18] Walumbwa F.O., Avolio B.J., Gardner W.L., Wernsing T.S., Peterson S.J. (2008). Authentic leadership: Development and validation of a theory-based measure. J. Manag..

[19] Avolio B.J., Gardner W.L. (2005). Authentic leadership development: Getting to the root of positive forms of leadership. Lead. Q.

[20] Avolio B.J., Gardner W.L., Walumbwa F.O., Luthans F., May D.R. (2004). Unlocking the mask: A look at the process by which authentic leaders impact follower attitudes and behaviors. Lead. Q.

[21] Alilyyani B., Wong C.A., Cummings G. (2018). Antecedents, mediators, and outcomes of authentic leadership in healthcare: A systematic review. Int. J. Nurs. Stud..

[22] Laschinger H.K., Smith L.M. (2013). The influence of authentic leadership and empowerment on new-graduate nurses’ perceptions of interprofessional collaboration. J. Nurs. Adm..

[23] Valsania S.E., León J.A., Alonso F.M., Cantisano G.T. (2012). Authentic leadership and its effect on employees’ organizational citizenship behaviours. Psicothema.

[24] Morris J.T. (2014). The impact of authentic leadership and ethical firm culture on auditor behavior. J. Behav. Stud. Busin..

[25] Ausar K., Kang H.J., Kim J.S. (2016). The effects of authentic leadership and organizational commitment on turnover intention. Leader Organiz. Deve J..

[26] Oh J., Cho D., Lim D.H. (2018). Authentic leadership and work engagement: The mediating effect of practicing core values. Leader Organiz. Deve J..

[27] Maximo N., Stander M.W., Coxen L. (2019). Authentic leadership and work engagement: The indirect effects of psychological safety and trust in supervisors. SA J. Indust. Psych..

[28] Leroy H., Anseel F., Gardner W.L., Sels L. (2015). Authentic leadership, authentic followership, basic need satisfaction, and work role performance: A cross-level study. J. Manag..

[29] Qiu S., Alizadeh A., Dooley L.M., Zhang R. (2019). The effects of authentic leadership on trust in leaders, organizational citizenship behavior, and service quality in the Chinese hospitality industry. J. Hosp. Tour Manag..

[30] Banks G.C., McCauley K.D., Gardner W.L., Guler C.E. (2016). A meta-analytic review of authentic and transformational leadership: A test for redundancy. The lead Q.

[31] Gardner W., Avolio B.J., Walumbwa F.O. (2005). Authentic Leadership Theory and Practice: Origins, Effects and Development.

[32] Kernis M.H. (2003). Toward a conceptualization of optimal self-esteem. Psych. Inq..

[33] Gardner W., Avolio B., Luthans F., May D., Walumbwa F. (2005). “Can you see the real me?” A self-based model of authentic leader and follower development. Lead. Quart.

[34] Ribeiro N., Gomes D., Kurian S. (2018). Authentic leadership and performance: The mediating role of employees’ affective commitment. Soc. Responsib. J..

[35] von Elm E., Altman D.G., Egger M., Pocock S.J., Gøtzsche P.C., Vandenbroucke J.P., STROBE Initiative (2008). The Strengthening the Reporting of Observational Studies in Epidemiology (STROBE) statement: Guidelines for reporting observational studies. J. Clin. Epidemiol..

[36] Avolio B.J., Gardner W.L., Walumbwa F.O. Authentic Leadership Questionnaire. www.mindgarden.com.

[37] Norman S.M., Avolio B.J., Luthans F. (2010). The impact of positivity and transparency on trust in leaders and their perceived effectiveness. Lead. Q.

[38] Rodwell J.J., Kienzle R., Shadur M.A. (1998). The relationships among work-related perceptions, employee attitudes, and employee performance: The integral role of communication. Hum. Resour. Manag..

[39] Al-Dossary R.N. (2022). Leadership Style, Work Engagement and Organizational Commitment Among Nurses in Saudi Arabian Hospitals. J. Healthc Lead..

[40] Alilyyani B., Kerr M., Wong C., Wazqar D. (2022). A Psychometric Analysis of the Nurse Satisfaction with the Quality of Care Scale. Healthc.

[41] Baker O.G., Alshehri B.D. (2020). The relationship between job stress and job satisfaction among Saudi nurses: A cross-sectional study. Nurse Med. J. Nurs..

[42] Almodibeg B.A., Smith H. (2021). A cross-sectional survey to explore the prevalence and causes of occupational burnout syndrome among perioperative nurses in Saudi Arabia. Nurs. Open.

[43] Wong C.A., Spence Laschinger H.K., Cummings G.G. (2010). Authentic leadership and nurses’ voice behaviour and perceptions of care quality. J. Nurs. Manag..

[44] Wong C.A., Giallonardo L. (2013). Authentic leadership and nurse-assessed adverse patient outcomes. J. Nurs. Manag..

[45] Alkaabi O., Wong C. (2019). Relationships among authentic leadership, manager incivility and trust in the manager. Lead. Health Serv..

[46] Iqbal S., Farid T., Khan M.K., Zhang Q., Khattak A., Ma J. (2020). Bridging the gap between authentic leadership and employees communal relationships through trust. Int. J. Environ. Res. Public Health.

[47] Agote L., Aramburu N., Lines R. (2016). Authentic leadership perception, trust in the leader, and followers’ emotions in organizational change processes. J. Appl. Behav. Sci..

[48] Wong C.A., Laschinger H.K. (2013). Authentic leadership, performance, and job satisfaction: The mediating role of empowerment. J. Advan. Nurs..

[49] Wong C.A., Cummings G.G. (2009). The influence of authentic leadership behaviors on trust and work outcomes of health care staff. J. Lead. Stud..

[50] Malik N. (2018). Authentic leadership–an antecedent for contextual performance of Indian nurses. Pers. Rev..

[51] Wang H.U., Sui Y., Luthans F., Wang D., Wu Y. (2014). Impact of authentic leadership on performance: Role of followers’ positive psychological capital and relational processes. J. Organ. Behav..

[52] Schwepker C.H., Schultz R. (2013). The impact of trust in manager on unethical intention and customer-oriented selling. J. Bus. Ind. Mark.

